# Cryopreservation of human vascular umbilical cord cells under good manufacturing practice conditions for future cell banks

**DOI:** 10.1186/1479-5876-10-98

**Published:** 2012-05-16

**Authors:** Bianca Polchow, Kati Kebbel, Gerno Schmiedeknecht, Anne Reichardt, Wolfgang Henrich, Roland Hetzer, Cora Lueders

**Affiliations:** 1 German Heart Institute Berlin, Department of Cardiothoracic and Vascular Surgery, Laboratory for Tissue Engineering, Augustenburger Platz 1, 13353, Berlin, Germany; 2 Fraunhofer Institute for Cell Therapy and Immunology IZI, Department of Cell Engineering/GMP, Perlickstr. 1, 04103, Leipzig, Germany; 3 Charité-Universitätsmedizin Berlin, Department of Obstetrics, Augustenburger Platz 1, 13353, Berlin, Germany; 4 Laboratory for Tissue Engineering German Heart Institute Berlin, Augustenburger Platz 1, Berlin, D-13353, Germany

**Keywords:** Vascular human umbilical cord cells, Good manufacturing practice (GMP), Cryopreservation, Cell banking

## Abstract

**Background:**

*In vitro* fabricated tissue engineered vascular constructs could provide an alternative to conventional substitutes. A crucial factor for tissue engineering of vascular constructs is an appropriate cell source. Vascular cells from the human umbilical cord can be directly isolated and cryopreserved until needed. Currently no cell bank for human vascular cells is available. Therefore, the establishment of a future human vascular cell bank conforming to good manufacturing practice (GMP) conditions is desirable for therapeutic applications such as tissue engineered cardiovascular constructs.

**Materials and methods:**

A fundamental step was the adaption of conventional research and development starting materials to GMP compliant starting materials. Human umbilical cord artery derived cells (HUCAC) and human umbilical vein endothelial cells (HUVEC) were isolated, cultivated, cryopreserved (short- and long-term) directly after primary culture and recultivated subsequently. Cell viability, expression of cellular markers and proliferation potential of fresh and cryopreserved cells were studied using trypan blue staining, flow cytometry analysis, immunofluorescence staining and proliferation assays. Statistical analyses were performed using Student’s t-test.

**Results:**

Sufficient numbers of isolated cells with acceptable viabilities and homogenous expression of cellular markers confirmed that the isolation procedure was successful using GMP compliant starting materials. The influence of cryopreservation was marginal, because cryopreserved cells mostly maintain phenotypic and functional characteristics similar to those of fresh cells. Phenotypic studies revealed that fresh cultivated and cryopreserved HUCAC were positive for alpha smooth muscle actin, CD90, CD105, CD73, CD29, CD44, CD166 and negative for smoothelin. HUVEC expressed CD31, CD146, CD105 and CD144 but not alpha smooth muscle actin. Functional analysis demonstrated acceptable viability and sufficient proliferation properties of cryopreserved HUCAC and HUVEC.

**Conclusion:**

Adaptation of cell isolation, cultivation and cryopreservation to GMP compliant starting materials was successful. Cryopreservation did not influence cell properties with lasting impact, confirming that the application of vascular cells from the human umbilical cord is feasible for cell banking. A specific cellular marker expression profile was established for HUCAC and HUVEC using flow cytometry analysis, applicable as a GMP compliant quality control. Use of these cells for the future fabrication of advanced therapy medicinal products GMP conditions are required by the regulatory authority.

## Background

Currently, cardiovascular diseases are the leading causes of global death and disability. An estimated 17.3 million people died from cardiovascular diseases in 2008 and 23.6 million people are predicted to die from them in 2030 [1]. Synthetic, metallic and biological grafts and valves are frequently and successfully used as implants in cardiac surgery, improving the quality of life for many patients. However, lower compliance of synthetic materials [2], higher thrombogenicity rates of metallic materials [3] and limited durability of biological materials [4,5] are undesirable limitations of these materials. Tissue engineered cardiovascular constructs, consisting of autologous cells that are incorporated in a biodegradable hemodynamic scaffold, could be a promising alternative to conventional replacements. Such constructs would be able to integrate, to regenerate and to grow - an aspect that is very attractive especially for pediatric patients affected by congenital cardiac disease such as heart valve failure [6]. Fabrication of functional implants requires extracellular matrix (ECM) producing cells and additional endothelial cells with antithrombogenic characteristics [7]. Further, cellular structures and cell functionality should be similar to those of native cardiovascular tissue, ensuring successful integration into the surrounding native tissue. Vascular cells from the human umbilical cord vein and arteries, including endothelial cells, smooth muscle cells and fibroblasts, represent a promising cell source for cardiovascular tissue engineering. Previous studies have shown that cells from the human umbilical cord synthesize ECM proteins such as collagen types I and III [8,9], also present in heart valves [10,11]. Kadner et al. found that umbilical cord artery derived cells express cytoskeletal filament proteins such as alpha smooth muscle actin (ASMA) and fibroblastic vimentin and additionally show fibroblast like morphology. For this reason they were considered to be myofibroblasts [12], an intermediate cell type between fibroblast and smooth muscle cells [13,14]. A comparative study revealed that CD90, fibronectin and collagen type I expression of human umbilical cord artery derived cells is similar to that of pulmonary heart valve interstitial cells. Furthermore, the expression of endothelial nitric oxide synthase (eNOS) and von Willebrandt factor (vWF) of endothelial cells from the human umbilical cord vein (HUVEC) is similar to that of pulmonary heart valve endothelial cells [15], showing that vascular cells from the human umbilical cord resemble the native cellular structures and functionalities found in heart valves. These properties encouraged the application of vascular umbilical cord cells for tissue engineering of cardiovascular constructs i.e. heart valves or blood vessels. Lüders et al. first reported that fresh and cryopreserved cells isolated from umbilical cord arteries and veins showed similar properties due to cell morphology and proliferation potential [16]. Continuative studies confirmed excellent proliferation and ECM formation properties *in vitro*, proving that cryopreserved vascular cells from the umbilical cord are convenient for in *vitro* generation of functional heart valves and for establishing autologous human cell banks [17]. The human umbilical cord as a potential source of vascular cells possesses several advantages: after delivery, redundant cord tissue can be used for direct isolation of the cells from cord vessels and no additional surgical intervention is necessary for the patient. Further, sufficient amounts of cells can be obtained, because an ordinary human umbilical cord, usually 20–22 in. long [18], provides enough tissue. Additionally, isolated cells can be cryopreserved as needed for operation. After cryopreservation cells can be thawed, recultured and expanded for fabrication of cardiovascular constructs. Using innovative cell banking technologies, vascular cells from the human umbilical cord can be used as an individual cell pool for the patient’s lifetime.

For this purpose, a cell bank providing high quality vascular cells, produced by well controlled standardized methods, represents an initial step for future fabrication of cardiovascular constructs. Different types of cell banks already exist worldwide. Amongst others, cells and cell lines of animal or human origin are supplied by the European Collection of Cell Cultures, established in 1984. The first successful umbilical cord blood transplantation, performed by Elianne Gluckman in 1988 led to the establishment of public cord blood banks worldwide [19]. Today, more than 100 active cord blood banks exist with more than 400,000 cord blood units stored for unrelated use [20]. The UK Stem Cell Bank is one of the most prominent non-profit stem cell banks, storing human adult and fetal stem cell lines [21]. Currently, no public cell bank exists for storing human vascular cells, even though the demand for primary cells in cellular therapy has recently increased. Following rapid development in the fields of biology, biotechnology and medicine, and the development of innovative techniques to examine diseases on the molecular and cellular level, single genes and cells are applied to treat different diseases. These so called “advanced therapy medicinal products” (ATMP) strongly differ from the medicinal products for human use available so far. Therefore, the new guideline “European Commission 1394/2007/EC” was created to regulate the marketing authorization of ATMP for innovative therapies [22]. ATMP comprise somatic cell therapy medicinal products, gene therapy medicinal products and tissue engineered medicinal products [23,24]. Concerning their first described future medical application, vascular cells from umbilical cord can be classified as ATMP which is of importance for the establishment of a cell bank consisting of these cells. Fabrication of cell-based medicinal products for clinical trials should take place in compliance with the principles of GMP, formulated in Commission Directive 2003/94/EC [25]. Safety aspects represent one of the main concerns in GMP production and refer to cell origin, the choice of starting materials, the manufacturing process itself and the associated quality controls [26]. Therefore, the origin and fabrication process of all starting materials should be adequately defined and well documented. The use of animal derived reagents such as fetal calf serum (FCS) should be avoided because of an increased risk of undesirable immunological responses in the recipient and a risk of the transmissible spongiform encephalopathy diseases. Furthermore, the fabrication process of the cell-based product should be designed in depth and validated to ensure product reproducibility and efficiency. To estimate the overall risk of a cell-based medicinal product, the following criteria have to be followed: autologous or allogenic origin, the ability of the cells to proliferate and the level of cell manipulation, including *in vitro* expansion, differentiation and cryopreservation.

In the recent study strategies for the establishment of a human vascular cell bank corresponding to the GMP guidelines were developed, to ensure essential quality and identity of the desired final cell-based medicinal product. The *in vitro* fabrication process for a cell bank was carefully designed by creating a flow chart of the entire process starting from 1. human umbilical cord tissue, 2. cell isolation, 3. cell cultivation in terms of primary culture, 4. cell harvest, leading to the final cell product prepared for 5. cryopreservation. The aims of the study involved consequent adaption from research and development (R&D) materials to GMP compliant starting materials concerning the cell isolation, cultivation and cryopreservation process. Furthermore, especially the influence of long term cryopreservation should be studied to control if the cells are applicable for future long term storage in GMP-certified cell banks. To satisfy requirements of GMP, quality controls should be established by performing extensive phenotypic and functional cell analysis.

As a long-term objective, the human vascular cell bank could supply cells for individual autologous or allogenic therapeutic applications such as the fabrication of tissue engineered cardiovascular constructs.

## Materials and methods

### Adaption to GMP compliant starting materials

To establish protocols of cell isolation, cultivation and cryopreservation conforming to GMP, adequate GMP grade starting materials had to be identified at first. Consequently, already established R&D protocols were remodelled concerning the starting materials. A number of R&D substances such as fetal calf serum (FCS) were completely replaced with agents conforming to GMP such as human AB serum (HS), because some of the R&D substances used so far were not available in GMP grade (Table 1). PBS and cell culture media were not replaced with regard to the GMP certified fabrication of the solutions (manufacturers’ instructions).

**Table 1:** Replacement of R&D starting materials to GMP compliant starting materials

	**R&D starting materials**	**GMP starting materials**
Enzymes	Dispase II (Roche Diagnostics, Mannheim, Germany)	Collagenase NB6 (Serva, Heidelberg, Germany)
Serum	Fetal calf serum (Biochrom AG, Berlin, Germany)	Human serum (pooled AB serum, Centre for Clinical Transfusion Medicine Tuebingen, Germany)
Supplements	Penicillin/Steptomycin (Invitrogen, Carlsbad, USA) Heparin (Sigma, St. Louis, USA) Basic fibroblast growth factor (Sigma, St. Louis, USA)	Gentamicin (PAA, Pasching, Austria) Heparin (pharmaceutical grade, Ratiopharm, Ulm, Germany) Basic fibroblast growth factor (clinical grade, Peprotech, Hamburg, Germany)
Cryopreservation	Fetal calf serum (Biochrom AG, Berlin, Germany) Dimethyl sulfoxide (Sigma, St. Louis, USA)	Human serum albumin (20 %, pharmaceutical grade, Baxter, Unterschleißheim, Germany) Dimethyl sulfoxide (pharmaceutical grade, Charité- Universitätsmedizin Berlin, Germany, product of the dispensary)

### Cell isolation and cultivation

Human umbilical cords were obtained from the Department of Obstetrics of the Charité Universitätsmedizin Berlin. The parents were informed about the objectives of the research project and after receiving the signed consent form from parents, the cords were processed after delivery. The research was carried out according to the principles of the Declaration of Helsinki and the study was approved by the Ethical Review Board at the Charité Universitätsmedizin Berlin, Humboldt University of Berlin, Germany (186/2001). According to the analysis of the adaption from R&D starting materials GMP compliant materials, human umbilical cords of similar length were divided in half and HUVEC as well as HUCAC were isolated, cultivated and harvested according to R&D and GMP conditions in parallel (Table 1).

After washing the cord with Dulbecco’s phosphate buffered saline (PBS) (Invitrogen, Carlsbad, USA) the vein was filled with 2.4 U/ml dispase II solution (Roche Diagnostics, Mannheim, Germany) and incubated for 30 min in a humidified atmosphere of 37°C and 5 % CO2 for isolation of HUVEC according to conventional R&D conditions. Under GMP conditions, 0.12 U/ml collagenase NB6 solution (Serva, Heidelberg, Germany) was injected into the vein and incubated for 20 min. In accordance with R&D conditions, collected HUVEC were cultured in Medium 199 (Invitrogen) supplemented with 10 % FCS (Biochrom AG, Berlin, Germany), 1 % penicillin/streptomycin (Invitrogen), 5 U/ml heparin (Sigma, St. Louis, USA) and 10 ng/ml basic fibroblast growth factor (bFGF, Sigma) at 37°C in a 5 % CO2 atmosphere. To achieve conditions conforming to GMP, Medium 199 was supplemented with 10 % HS (pooled AB serum, Centre for Clinical Transfusion Medicine Tuebingen, Germany), 1 % gentamicin (PAA, Pasching, Austria), 5 U/ml heparin (pharmaceutical grade, Ratiopharm, Ulm, Germany) and 10 ng/ml bFGF (Peprotech, Hamburg, Germany). The following day, the HUVEC were washed with PBS to remove all non-adherent cells. At confluence, the cells were trypsinized (0.05 % trypsin/0.02 % EDTA, Invitrogen) and then cryopreserved after primary culture.

HUCAC were isolated by mechanical excision. Cord arteries were excised out of the ambient tissue, minced into 1 mm pieces and placed in polystyrene dishes (Sarstedt, Nuembrecht, Germany) allowing the cells to grow out of the arterial wall (explant culture). Under R&D conditions, sections were cultured in Dulbecco’s modified Eagle’s medium (DMEM, Invitrogen) supplemented with 20 % FCS and 1 % penicillin/ streptomycin (Invitrogen) in a humidified atmosphere of 37°C and 5 % CO2. To obtain GMP conditions, DMEM (Invitrogen) was supplemented with 5 % HS and 1 % gentamicin (PAA). After approximately 21 days, sufficient cell numbers were harvested and cryopreservation was initiated after primary culture.

### Cryopreservation and recultivation

Harvested HUCAC and HUVEC from primary culture (passage 0) isolated with GMP compliant starting materials were used for cryopreservation. A pellet of about 1.0 x 10^6^ cells was assimilated in 1 ml freezing media, consisting of 90 % of a 20 % human serum albumin solution (HSA, Baxter, Unterschleißheim, Germany) and 10 % dimethyl sulfoxide (DMSO, Charité-Universitätsmedizin Berlin, Germany), rapidly transferred to a cryovial (Nunc, Langenselbold, Germany) and stored in an isopropanol bath freezing container (Nalgene, Rochester, USA) at – 80°C for 24 hours. Isopropanol ensures cell cooling at a steady freezing rate of −1°C/min down to −80°C. Finally, cells were placed in the vapour phase of liquid nitrogen for short-term (7 days) and long-term (1 year) storage. After the cryopreservation period the cells were rapidly thawed by immersing the vials in a water bath for 2 min at 37°C. Cells were resuspended in culture medium, centrifuged at 360 x g for 6 min to remove residual DMSO, and recultivated in a humidified atmosphere of 37°C and 5 % CO2 for subcultivation. Cell culture medium was replaced every 2–3 days.

### Cell viability

Viability of short-term cryopreserved HUCAC (group A: n = 5) and HUVEC (group A: n = 4) and long- term cryopreserved HUCAC (group B: n = 4) and HUVEC (group B: n = 5) from primary cultures was studied after the thawing process and in the first passage of recultivation. Non-cryopreserved fresh cultivated HUCAC (n = 5) and HUVEC (n = 4) were analyzed in parallel as control groups. The cells were resuspended in appropriate cell culture medium and diluted with trypan blue solution (1:1). Unstained cells were counted as viable cells by using a hemacytometer. Cell viability is calculated by the number of viable cells divided by the number of total cells in percent.

### Proliferation potential

Proliferation potential of short-term cryopreserved HUCAC (group A: n = 3) and HUVEC (group A: n = 4) and long-term cryopreserved HUCAC (group B: n = 3) and HUVEC (group B: n = 5) was quantified using a colorimetric water soluble tetrazolium assay (WST-1, Roche Diagnostics, Mannheim, Germany). Non-cryopreserved fresh cultivated HUCAC (n = 3) and HUVEC (n = 4) were analyzed in parallel as control groups. The cells were seeded in 96-well plates at a concentration of 2.0 x 10^3^ cells/well and cultivated in appropriate cell culture medium at 37°C in a 5 % CO2 atmosphere. Culture medium was changed every 2–3 days. On days 0, 1, 2, 3, 4 and 7 10 μl WST-1 solution was added to 100 μl sample supernatant (quintuplicates) and incubated for 2 h at 37°C. This procedure was repeated for cells of passages 2–5. The optical density (OD) of supernatants was measured against a background control as blank (culture medium without cells) at 450 nm using an absorbance microplate reader (Biotek, Bad Friedrichshall, Germany). Reference wavelength of 630 nm was subtracted from averaged OD values measured at 450 nm. Growth curves for direct and quantitative analysis of proliferation were created. The OD values obtained were correlated to the number of living cells, i.e. the higher the OD values, the higher the cell number.

### Flow cytometry analysis

Surface and intracellular marker expression of fresh cultivated HUCAC (control group: n = 3) and HUVEC (control group: n = 3), short-term cryopreserved HUCAC (group A: n = 4) and HUVEC (group A: n = 4) and long-term cryopreserved HUCAC (group B: n = 4) and HUVEC (group B: n = 4) was detected by flow cytometry analysis. Single cell suspensions of 1.4 x 10^5^ cells in 100 μl 2 % fetal bovine serum/PBS were prepared from HUCAC and HUVEC of primary culture (passage 0), passage 3 and 4. Single- and multi-color stainings were performed using fluorescein isothiocyanate (FITC), phycoerythrin (PE) or allophycocyanin (APC) conjugated mouse monoclonal antibodies. Corresponding isotype immunoglobulins were used as negative controls for each antibody.

First multi-color staining was performed by incubating HUCAC with anti-human CD44-FITC (1:20, Beckman Coulter, Krefeld, Germany), anti-human CD166-PE (2.5 μg/ml, R&D Systems, Minneapolis, USA) and anti-human CD105 –APC (2.5 μg/ml, R&D Systems) for 45 min at 4°C after blocking with Fc-blocking reagent (Miltenyi Biotec, Teterow, Germany). Second multi-color staining was performed by incubating HUCAC with anti-human CD90-FITC (1:20, Beckman Coulter), anti-human CD29-APC (1:20, BD Biosciences, Heidelberg, Germany) and anti-human CD73-PE (1:20, BD Biosciences) for 45 min at 4°C. Single-color staining to intracellular human ASMA (PE-conjugated, 2.5 μg/ml, R&D Systems) was performed for 45 min at room temperature (RT) after fixation and permeabilization with Intra Präp Kit (Beckman Coulter).

Single-color staining was performed by incubating HUVEC with anti-human CD146-APC (10 μg/ml, Miltenyi Biotec) for 10 min at 4°C. Multi-color staining to anti-human CD105-APC (2.5 μg/ml, R&D Systems) and anti-human CD31-FITC (20 μg/ml, BD Biosciences) was performed for 45 min at 4°C. After fixation and permeabilization with the Intra Präp Kit (Beckman Coulter) anti-human CD144-PE (20 μg/ml, Beckman Coulter) was added for 15 min at RT. Accordingly, the cells were washed twice with PBS, fixed in 2 % paraformaldehyde solution and analyzed by using a flow cytometer (Beckman Coulter).

### Immunofluorescence staining

Expression of cellular marker molecules and production of different ECM proteins were qualitatively analyzed by indirect immunofluorescence staining. Fresh cultivated and cryopreserved HUCAC (each n = 3) and HUVEC (each n = 3) were seeded onto glass coverslips and fixed with ice-cold methanol/acetone solution (1:1) after reaching confluence. HUCAC were incubated with primary monoclonal mouse antibodies against human CD90 (4 μg/ml, Dianova, Hamburg, Germany), human CD105 (10 μg/ml, BD Biosciences, Heidelberg, Germany), human CD29 (625 ng/ml, BD Biosciences), human CD44 (5 μg/ml, BD Biosciences), human fibronectin (5 μg/ml, BD Biosciences) and with polyclonal rabbit antibody against human ASMA (25 μg/ml, Abcam, Cambridge, UK), human collagen types I & III (0.5 μg/ml, Acris Antibodies, Herford, Germany), human elastin (1:50, Calbiochem, Darmstadt, Germany) and smoothelin (3.6 μg/ml, Abcam) for 3 h at 4°C. HUVEC were incubated with monoclonal mouse antibodies against human CD31 (1:50, Sigma, St.Louis, USA), human CD144 (10 μg/ml, BD Biosciences), human CD146 (25 μg/ml, BD Biosciences), human vWF (8 μg/ml, Millipore, Billerica, USA) and with polyclonal rabbit antibody against human eNOS (12.5 μg/ml, BD Biosciences) and ASMA (25 μg/ml, Abcam) for 3 h at 4°C. After incubation with primary antibodies, cells were washed three times with PBS, followed by incubation with appropriate FITC/TRITC conjugated secondary anti-mouse/rabbit antibodies (1:400, Invitrogen, Carlsbad, USA) for 2 h at RT. Fixed cross sectioned umbilical cord arteries were used to localize HUCAC and served as controls by staining with monoclonal mouse anti-human CD90 (4 μg/ml, Dianova), anti-human fibronectin (5 μg/ml, BD Biosciences) and with polyclonal rabbit anti-human ASMA (25 μg/ml, Abcam), anti-human collagen types I & III (0.5 μg/ml, Acris Antibodies), anti-human elastin (1:50, Calbiochem) for 3 h at 4°C. Cross sections of umbilical cord vein served also as controls and were incubated with monoclonal mouse anti-human CD31 (1:50, Sigma) and anti-human vWF (8 μg/ml, Millipore) for 3 h at 4°C to detect HUVEC. Subsequently, staining with corresponding secondary FITC/TRITC labelled anti-mouse/rabbit antibodies (Molecular Probes, Eugene, USA) was performed. Cell nuclei were stained with DAPI (Roche Diagnostics, Mannheim, Germany) for 10 min at RT and finally cells and cross sections were mounted with Mowiol mounting medium (Calbiochem).

### Immunohistochemical staining

Expression of cellular marker molecules and production of different ECM proteins was qualitatively analyzed by immunohistochemical staining. Stainings were automatically performed in a Bond-MAX stainer (Leica, Wetzlar, Germany). Fixed cross sectioned umbilical cord arteries were used to localize ECM proteins and served as controls by staining with monoclonal mouse anti-human collagen type I (0.5 μg/ml, Acris Antibodies, Herford, Germany), anti-human collagen type III (30 μg/ml, Innovative Diagnostics, Vienna, Austria) and anti-human elastin (1:100, Sigma, St.Louis, USA) for 30 min at RT after blocking with goat serum (10 %, Leica) for 10 min. Cross sections of umbilical cord vein, serving as controls, were incubated with monoclonal mouse anti-human CD31 (1:80, Innovative Diagnostics) and anti-human vWF (1:100, Dako, Hamburg, Germany) for 30 min to detect HUVEC after blocking with goat serum (10 %, Leica) for 10 min. Sections were rinsed with washing solution (10 %, Leica) and incubated with corresponding secondary alkaline phosphatase-labelled antibodies (Detection Kit, Leica) for 10 min. After rinsing with washing solution (10 %, Leica), enzyme substrate (Polymer AP Red Detection Kit, Leica) was added for 20 min. Counterstaining was performed for 3 min using hematoxyline staining solution (30 %, Thermo-Shandon, Frankfurt, Germany) to visualize cell nuclei. Finally, cross sections were dehydrated with 90 % and 100 % alcohol solution and subsequently embedded in mounting medium (Cytoseal XYL, Thermo Scientific, Waltham, USA).

### Differentiation potential

Differentiation potential of fresh cultivated HUCAC from passage 2 (n = 3) and 4 (n = 3) was studied using a multilineage method including analysis of osteogenic, chondrogenic and adipogenic differentiation potential [27]. Bone marrow derived mesenchymal stem cells (MSC) from passage 2 (n = 1) and 4 (n = 1) were analyzed in parallel, serving as positive controls. Analysis of differentiation potential was performed according to R&D conditions.

#### *Osteogenesis*

Cells were stimulated with osteogenic medium, consisting of DMEM (1 g/l Glucose, Invitrogen, Carlsbad, USA) supplemented with 5 % FCS (Biochrom AG, Berlin, Germany), 1 % penicillin/streptomycin (10,000 U/10,000 μg/ml, Invitrogen), 100 nM dexamethasone (Sigma, St.Louis, USA), 10 mM ß-glycerophosphate (Sigma), 0.17 mM L-ascorbic-acid (Sigma), for at least 28 days.

Medium was changed every 2–3 days. To detect the mineralization process, calcium accumulations were stained by the Von Kossa method. Cells were fixed with iced methanol for 30 min at −20°C, washed with sterile water and treated with 5 % silver nitrate solution (Sigma) for 30 min at RT with absence of light. Finally, 5 % sodium bicarbonate solution (Sigma) supplemented with 1 % formaldehyde (37 %, Sigma, Germany) was used to detect calcium accumulations. The surface enzyme alkaline phosphatase, whose activity is increased on osteoblasts [28], was detected by BCIP/NBT staining solution (Roche Diagnostics, Mannheim, Germany). The fixed cells were stained for 10 min at RT by absence of light and washed with distilled water.

#### *Adipogenesis*

Adipogenic differentiation was induced by incubating the cells with DMEM (4.5 g/l glucose, Invitrogen) supplemented with 10 % FCS (Biochrom AG), 1 % penicillin/streptomycin (10,000 U/10,000 μg/ml, Invitrogen), 0.01 mg/ml insulin **(**Insuman Comb 25, Sanofi Aventis-40 I.E./ml, Germany), 0.2 mM indomethacine (Sigma), 0.5 mM 3-isobutyl-1-methyl-xanthine (IBMX) (Sigma), 1 μM dexamethasone (Sigma) for at least 25 days. The stimulation process was performed in 3–4 cycles. One cycle lasted 5 days, in which the cells were stimulated with adipogenic medium for the first 3 days and for the remaining 2 days with adipogenic medium without stimulating factors. To detect fat cell-specific enhanced lipid vacuoles the cells were stained with 0.5 % Oil Red O solution (Sigma), a liposoluble dye which stains triglycerides.

#### *Chondrogenesis*

High dense micromass pellet cultures composed of agglomerated HUCAC were generated in 15 ml centrifuge tubes (0.4 x 10^6^ cells/tube) and stimulated with serum-free DMEM (4.5 g/l glucose, Invitrogen) culture medium supplemented with 1 % penicillin/streptomycin (10000 U/10000 μg/ml, Invitrogen), 1 % ITS + 1 supplements (Sigma), 100 nM dexamethasone (Sigma), 1 mM sodium pyruvate (Invitrogen); 0.17 mM L-ascorbic-acid (Sigma); 0.35 mM proline (Sigma) and 10 ng/ml TGFß-3 (Promocell, Heidelberg, Germany) for at least 28 days. Medium was changed every 2–3 days. To detect cartilage-specific collagen type II, cross-sectioned micromass cell pellets (HUCAC) were stained with rabbit polyclonal anti-human collagen II (1:40, Leica, Wetzlar, Germany) and specific proteoglycans (aggrecan) were visualized by Alcian PAS staining.

All staining analyses were performed using bright field light microscopy.

### Statistical analysis

All experiments were performed at least in triplicate. Results were expressed as mean values ± standard deviation. Statistical analysis was performed by using Graph Pad Prism (Version 5.01). Comparisons between recultivated, cryopreserved cells (group A and B) and fresh cultivated cells (control group) were done using Student’s t-test. Differences were considered statistically significant at P < 0.05.

## Results

### Adaption to GMP compliant starting materials

**Figure 1:**
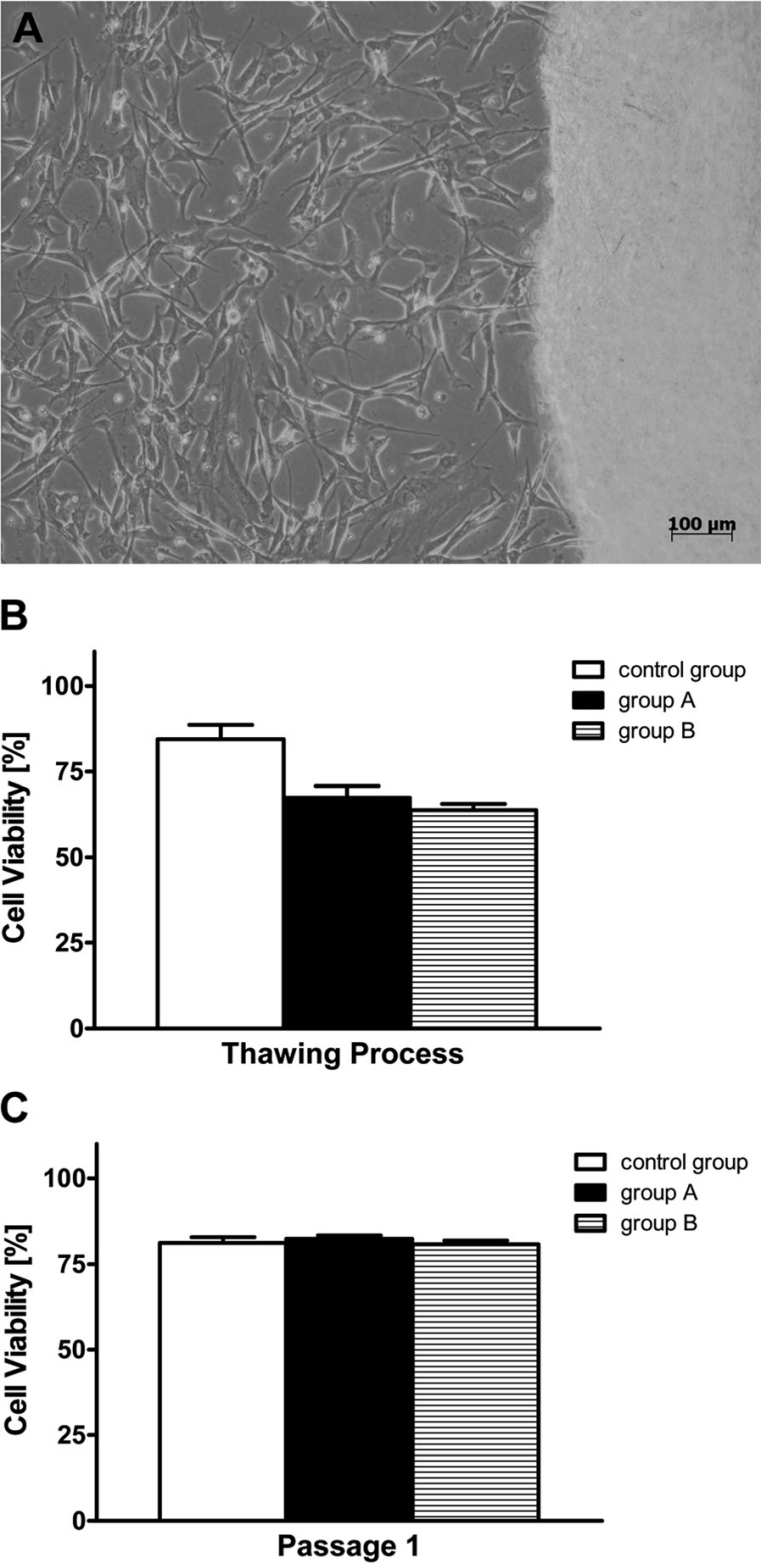
**Outgrowth and viability of human umbilical cord artery derived cells (HUCAC).A**) HUCAC isolated by mechanical excision grew out of single cord artery pieces (explant culture) after approximately 21 days and demonstrated robust spindle-shaped morphology in primary cultures (passage 0). Cells from primary cultures were used for further cryopreservation studies. Viability of short-term (group A, n = 5) and long-term (group B, n = 4) cryopreserved cells from primary cultures (passage 0) was studied directly after **B**) thawing and **C**) in passage 1 of recultivation using trypan blue staining assay. By comparison, non-cryopreserved fresh cells (n = 5) from primary cultures and passage 1 were analyzed in parallel as control group. The number of viable cells divided by the number of total cells results in cell viability (%).

**Figure 2:**
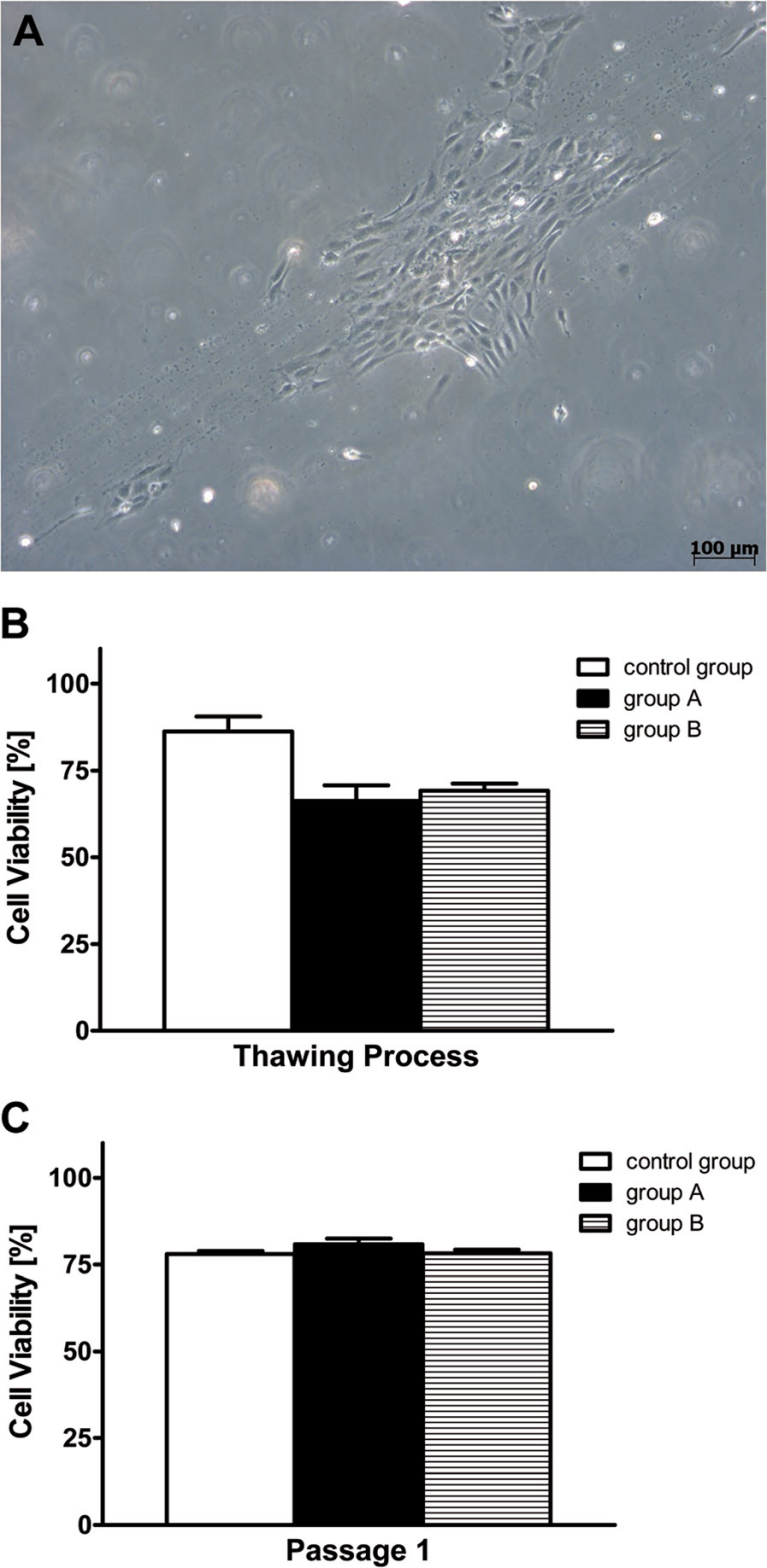
**Growth colony formation and viability of human umbilical vein endothelial cells (HUVEC).A**) Enzymatically isolated HUVEC first grew in scattered colonies and demonstrated typical rounded, cobblestone-like morphology. After reaching confluence in primary cultures (passage 0) cells were used for further cryopreservation studies. Viability of short-term (group A, n = 4) and long-term (group B, n = 5) cryopreserved cells from primary cultures (passage 0) was studied directly after **B**) thawing and **C**) in passage 1 of recultivation using trypan blue staining assay. By comparison, non- cryopreserved fresh cells (n = 4) from primary cultures and passage 1 were analyzed in parallel as control group. The number of viable cells divided by the number of total cells results in cell viability (%).

Adaption from conventional R&D starting materials to GMP compliant starting materials was studied concerning cell isolation and subsequent cell cultivation (primary culture). HUCAC quickly grew out of single explant pieces and showed robust spindle-shaped morphology in primary cultures (Figure 1A). HUVEC demonstrated a rounded, cobblestone-like morphology (Figure 2A). Comprehensive homogenous confluence arising from initial scattered colonies (Figure 2A) was observed in primary culture. No significant morphological differences were observed for HUCAC and HUVEC of primary cultures isolated with either R&D or GMP protocols.

Using R&D protocol, 6.8 x 10^6^ ± 2.2 x 10^6^ HUCAC (n = 4) and 3.0 x 10^6^ ± 0.6 x 10^6^ HUVEC (n = 4) were obtained from primary cultures. Similar cell numbers were harvested for each cell type using the GMP protocol: HUCAC (n = 4): 7.0 x 10^6^ ± 1.3 x 10^6^ and HUVEC (n = 4): 3.8 x 10^6^ ± 1.2 x 10^6^.

### Cell viability

Cell viability of short-term cryopreserved (group A) and long-term cryopreserved (group B) cells was directly measured after thawing and in passage 1 of recultivation using the trypan blue assay. Fresh cells were analyzed in parallel as a control group. After the thawing process, the viability of group A and B HUCAC was decreased to 67.4 ± 7.6 % and 63.8 ± 3.6 % and significantly lower (P = 0.011, P = 0.015) than the viability of fresh cultivated cells (control group) from primary cultures (84.4 ± 9.4 %), shown in Figure 1B. Already in passage 1, cell viability of recultivated cryopreserved HUCAC from group A (82.4 ± 2.2 %) and B (80.7 ± 2.2 %) increased and was comparable to the viability of the control group (81.2 ± 3.7 %), represented in Figure 1C. No significant difference in cell viability was detected by comparing the control group with group A (P = 0.625) and the control group with group B (P = 0.694) in passage 1.

Viability of HUVEC from group A and B was about 66.3 ± 8.8 % and 69.2 ± 4.6 %, directly determined after the thawing process (Figure 2B). Fresh cultivated cells (control group) demonstrated higher viabilities of about 86.3 ± 8.6 %. Thus, significant differences in cell viability were detected by comparing group A (P = 0.024) and B (P = 0.023) to the control group. Increased viabilities of HUVEC from group A and B were detected in passage 1 (80.7 ± 3.4 % and 78.2 ± 1.1 %), similar to the viability of control group cells (78.0 ± 1.8 %) (Figure 2C). Accordingly, comparing cell viability of the control group with group A (P = 0.295) and with group B (P = 0.815) no significant differences were detected.

### Proliferation potential

**Figure 3:**
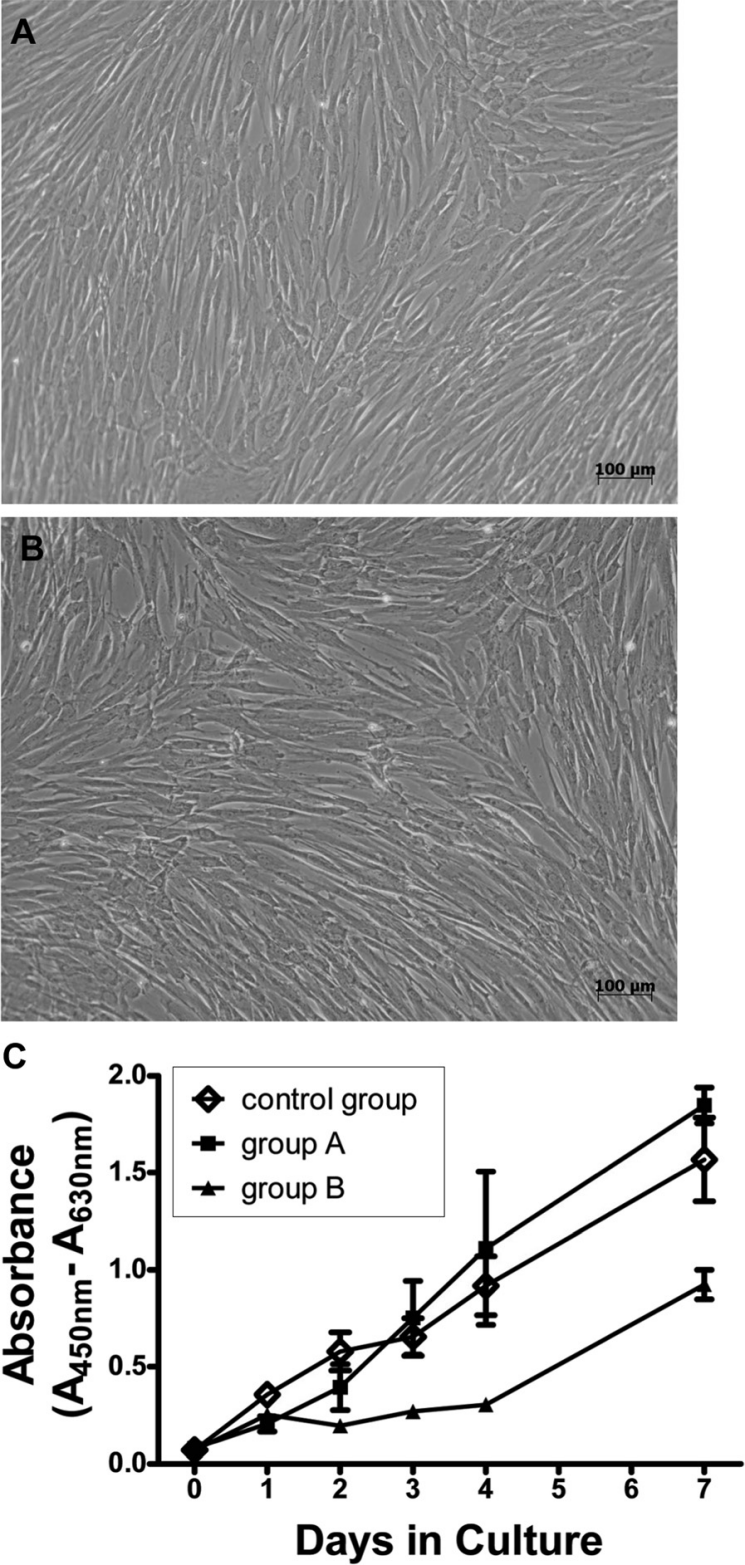
**Proliferation potential of human umbilical cord artery derived cells (HUCAC).** Significant morphological differences were not observed for **A**) fresh cultivated and **B**) cryopreserved HUCAC. **C**) Proliferation potential of recultivated short-term (group A, n = 3) and long-term (group B, n = 3) cryopreserved cells was quantified using a colorimetric WST-1 assay. By comparison, non- cryopreserved fresh cells (n = 3) were analyzed in parallel as a control group (C). The optical density (OD) was measured at 450 nm and reference wavelength of 630 nm was subtracted. OD values were correlated to the number of living cells and higher OD values meant a higher cell number. Morphology and proliferation potential are exemplarily shown for cells of passage 3.

**Figure 4:**
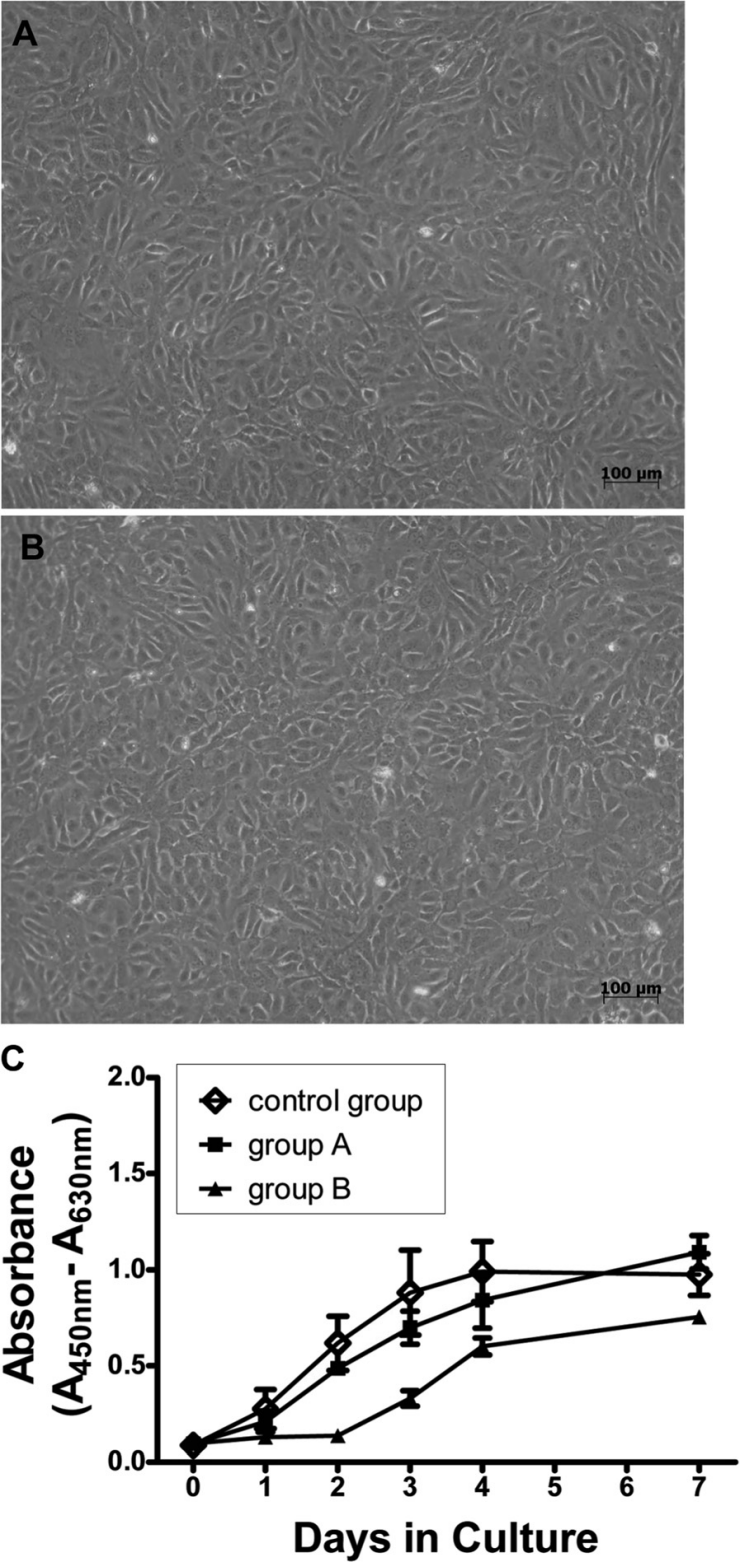
**Proliferation potential of human umbilical vein endothelial cells (HUVEC).** Significant morphological differences were not observed for **A**) fresh cultivated and **B**) cryopreserved HUVEC. **C**) Proliferation potential of recultivated short-term (group A, n = 4) and long-term (group B, n = 5) cryopreserved cells was quantified using a colorimetric WST-1 assay (C). By comparison, non- cryopreserved fresh cells (n = 4) were analyzed in parallel as a control group. The optical density (OD) was measured at 450 nm and reference wavelength of 630 nm was subtracted. OD values were correlated to the number of living cells and the higher OD values meant a higher cell number. Morphology and proliferation potential are exemplarily shown for cells of passage 3.

Proliferation potential of short-term cryopreserved (group A) and long-term cryopreserved (group B) cells was measured for several passages, using the WST-1 proliferation assay. Fresh cells were cultivated in parallel as a control group. A continuous increase in cell number was detected for all tested groups of HUCAC with maximal absorbance on day 7, exemplarily shown for passage 3 (Figure 3C). Related to initial absorbance (day 0), a 22.1-fold, 23.1-fold and 13.6-fold increase was detected for the control group, group A and group B on day 7 (Figure 3C). No significant difference in proliferation was detected for the control group and group A from day 0 (P = 0.421) to day 7 (P = 0.066) in culture. However, comparing group B HUCAC to the control group, a significant difference in proliferation was determined from day 1 (P = 0.022) to day 4 (P = 0.010), but not on day 7 (P = 0.059). After a short lag phase, increased absorbance was detected for all tested groups of HUVEC, starting from day 1 to day 7 with maximal absorbance on day 4 (control group) and day 7 (group A, B), exemplarily shown for passage 3 (Figure 4C). In relation to the initial absorbance value, a 11.2-fold, 11.5-fold and 7.7-fold increase was measured for control group, group A and group B. Comparing the proliferation potential of the control group and group A, no significant difference was detected from day 0 (P = 0.592) to day 7 (P = 0.182). However, the proliferation potential of HUVEC from group B was significantly lower than that of the control group from day 1 (P = 0.039) until day 3 (P = 0.021), but not on day 4 (P = 0.0543) or day 7 (P = 0.078).

Altogether, similar trends of growth curves were determined for recultivated cryopreserved (group A and B) and fresh cultivated (control group) HUCAC and HUVEC. Additionally, no significant morphological differences were observed for fresh cultivated HUCAC (Figure 3A) and cryopreserved HUCAC (Figure 3B) or for fresh cultivated HUVEC (Figure 4A) and cryopreserved HUVEC (Figure 4B), exemplarily shown for fresh cultivated and short-term cryopreserved cells of passage 3.

### Expression of cellular markers

**Figure 5:**
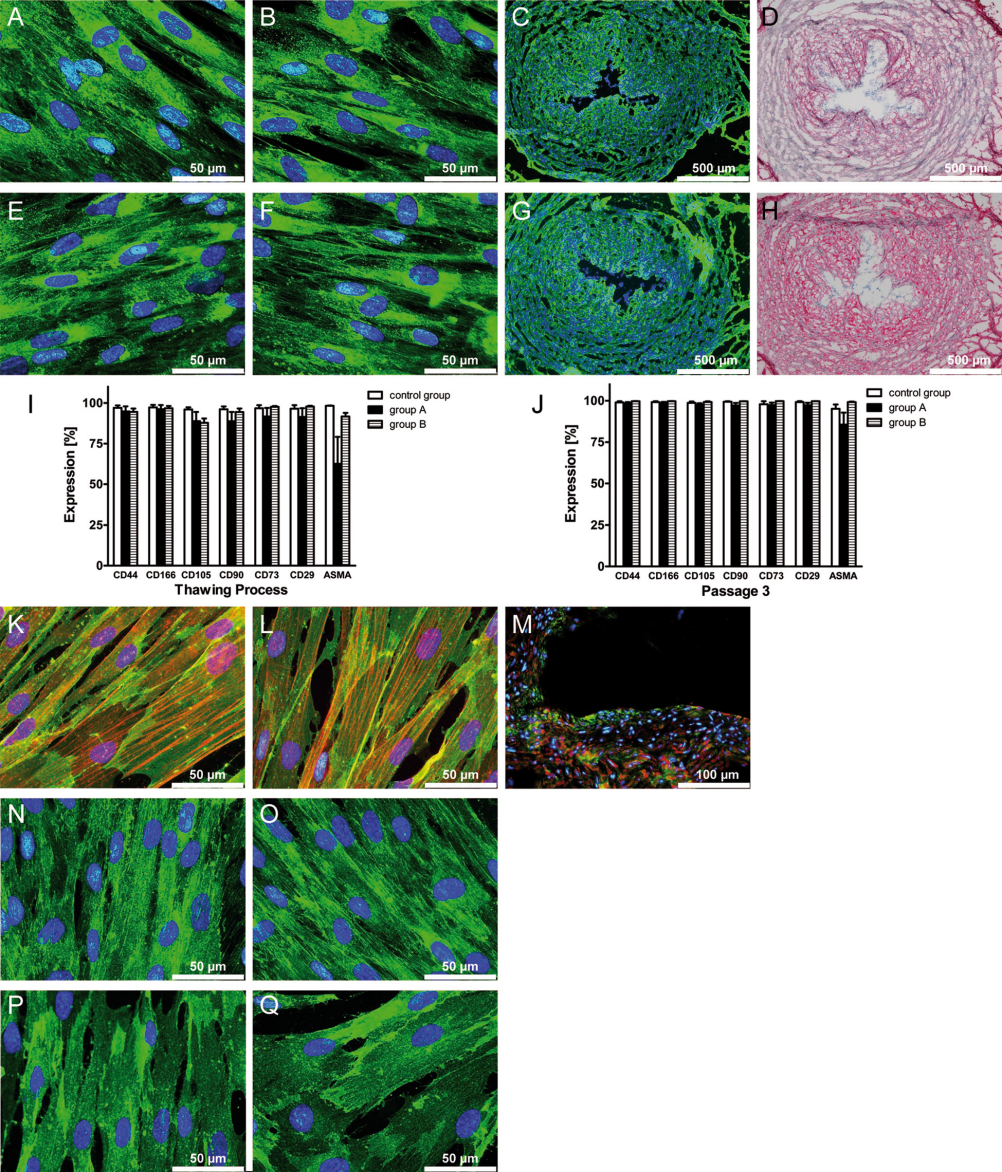
**Expression of cellular marker molecules and extracellular matrix (ECM) proteins by human umbilical cord artery derived cells (HUCAC).** Using indirect immunofluorescence staining, highly positive signals (green) were detected for **A**) collagen type I of fresh cultivated cells and **B**) collagen type I of cryopreserved cells, **E**) collagen type III of fresh cultivated cells and **F**) collagen type III of cryopreserved cells. The presence of **C**) collagen type I (green) and **G**) collagen type III (green) was shown in native human umbilical cord artery walls, serving as a control. Immunohistochemical staining verified the presence of **D**) collagen type I (red) and **H**) collagen type III (red) in native human umbilical cord artery walls. Using flow cytometry analysis, cellular marker expression of short-term (group A, n = 4) and long-term (group B, n = 4) cryopreserved cells from primary cultures (passage 0) was studied directly after **I**) thawing and **J**) in passage 3 of recultivation. By comparison, non-cryopreserved fresh cells (n = 3) from **I**) primary cultures and **J**) passage 3 were analyzed in parallel as a control group Using indirect immunofluorescence staining, highly positive signals (green) were detected for all cellular markers tested such as **K**) CD90 (green)/ alpha smooth muscle actin (ASMA) (red) of fresh cultivated cells and **L**) CD90 (green)/ ASMA (red) of cryopreserved cells, **N**) CD29 of fresh cultivated cells and **O**) CD29 of cryopreserved cells, **P**) CD105 of fresh cultivated cells and **Q**) CD105 of cryopreserved cells. Cell nuclei staining is pictured in blue, present in A-H and K-Q. All studies of marker expression are exemplarily shown for cells of passage 3.

Expression of cellular markers of short-term cryopreserved (group A) and long-term cryopreserved (group B) cells was studied after thawing and in passage 3 and 4 of recultivation by flow cytometry analysis and indirect immunofluorescence staining. Fresh cultivated cells from passage 0, 3 and 4 were studied in parallel as a control group. Results of flow cytometry analysis revealed that HUCAC are positive for ASMA and CD90 as well as for the markers of mesenchymal stem cells such as CD44, CD166, CD105, CD73 and CD29. Directly after thawing, cellular marker expression of cells from group A and B was decreased compared to expression levels of the control group (Figure 5 I). Especially, expression of the intracellular marker protein ASMA was reduced to levels of about 62.5 ± 33.3 % (group A) after the thawing process. Nevertheless, no significant difference in marker expression was detected for HUCAC from group A and B compared to the control group, regarding all tested markers (P > 0.05). Recultivated cells from group A and B demonstrated increased expression levels of cellular markers, nearly or completely achieving levels of markers expressed by fresh cultivated cells (control group), exemplarily shown for passage 3 in Figure 5 J. The difference in expression levels for all tested cellular markers was not significant for cells of group A and B compared to the control group (P > 0.05).

**Figure 6:**
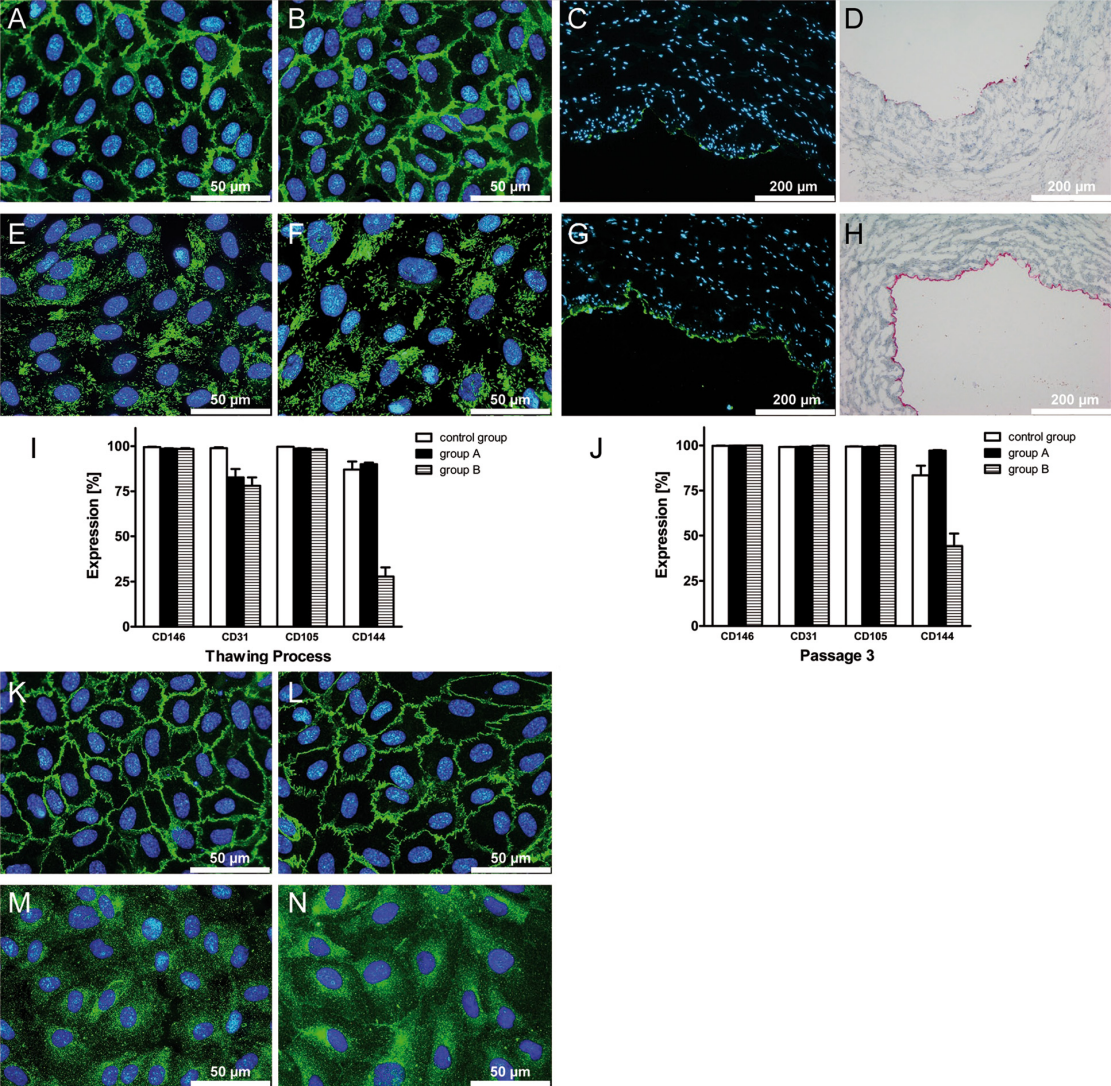
**Expression of cellular marker molecules by human umbilical vein endothelial cells (HUVEC).** Using indirect immunofluorescence staining, highly positive signals (green) were detected for **A**) CD31 of fresh cultivated cells and **B**) CD31 of cryopreserved cells, **E**) von Willebrandt factor (vWF) of fresh cultivated cells and **F**) vWF of cryopreserved cells. The presence of **C**) CD31 (green) and **G**) vWF (green) was detected in the endothelium of native human umbilical cord veins, serving as a control. Immunohistochemical staining verified the presence of **D**) CD31 (red) and **H**) vWF (red) in the endothelium of native human umbilical cord veins. Using flow cytometry analysis, cellular marker expression of short-term (group A, n = 4) and long-term (group B, n = 4) cryopreserved cells from primary cultures (passage 0) was studied directly after **I**) thawing and **J**) in passage 3 of recultivation. By comparison, non-cryopreserved fresh cells (n = 3) from I) primary cultures and J) passage 3 were analyzed in parallel as a control group. Using indirect immunofluorescence staining, highly positive signals (green) were also detected for the cellular markers **K**) CD144 of fresh cultivated cells and **L**) CD144 of cryopreserved cells, **M**) endothelial nitric oxide-synthase (eNOS) of fresh cultivated cells and **N**) eNOS of cryopreserved cells. Cell nuclei staining is pictured in blue, present in A-H and K-N. All studies of marker expression are exemplarily shown for cells of passage 3.

Endothelial cell specific markers such as CD146, CD31, CD105 were highly expressed by fresh cultivated HUVEC (control group). Lower value of expression was detected for CD144 (about 86.9 ± 7.8 %), shown in Figure 6 I. In comparison with the control group, expression of CD31 was decreased to levels of about 82.6 ± 9.4 % (group A) and 78.0 ± 10.3 % (group B), measured directly after thawing. Cells from group B demonstrated significantly decreased expression of CD144 (27.8 ± 11.5 %, P = 0.036), as well. However, expression of CD146 and CD105 remained stable for HUVEC from group A and B after thawing. In passage 3, almost all endothelial cell specific markers were still highly expressed by HUVEC from group A and B and achieved the expression levels of the control group (Figure 6 J). Hence, no significant difference in cellular marker expression was detected for group A and B compared to control group (P > 0.05). Only HUVEC from group B still demonstrated low expression of CD144, significantly different from the control group (P = 0.009).

Indirect immunofluorescence studies were performed to verify the results of flow cytometry analysis and are exemplarily shown for fresh cultivated (control group) and recultivated short-term cryopreserved (group A) HUCAC and HUVEC from passage 3. Intense positive signals were detected for HUCAC stained with anti-CD44 (data not shown), anti-CD105 (Figure 5 P, Q) and anti-CD29 (Figure 5 N, O). Double staining against CD90 and ASMA revealed that a single cell expresses both markers simultaneously (Figure 5 K, L).The presence of CD90 and ASMA was also detected in human umbilical cord artery walls (Figure 5 M). No difference in signal intensity was observed for HUCAC from the control group and group A. Smoothelin, a late differentiation marker of contractile smooth muscle cells, was not expressed by HUCAC (data not shown). ECM proteins such as collagen I, III, fibronectin (data not shown) and elastin (data not shown) were expressed by HUCAC from the control group and group A with no difference in signal intensity (Figure 5 A, B, E, F). These proteins were also detected in native human umbilical cord arteries represented in Figure 5 C, D, G, H.

Positive signals were detected for CD31 (Figure 6 A, B), vWF (Figure 6 E, F), CD144 (Figure 6 K, L), CD146 (data not shown) and eNOS (Figure 6 M, N) with no evident difference in fluorescence intensity between HUVEC from the control group and group A. Presence of CD31 and vWF was also detected in endothelium of the human umbilical vein (figure C, D and G, H). HUVEC were negative for ASMA (data not shown).

### Analysis of differentiation potential

**Figure 7:**
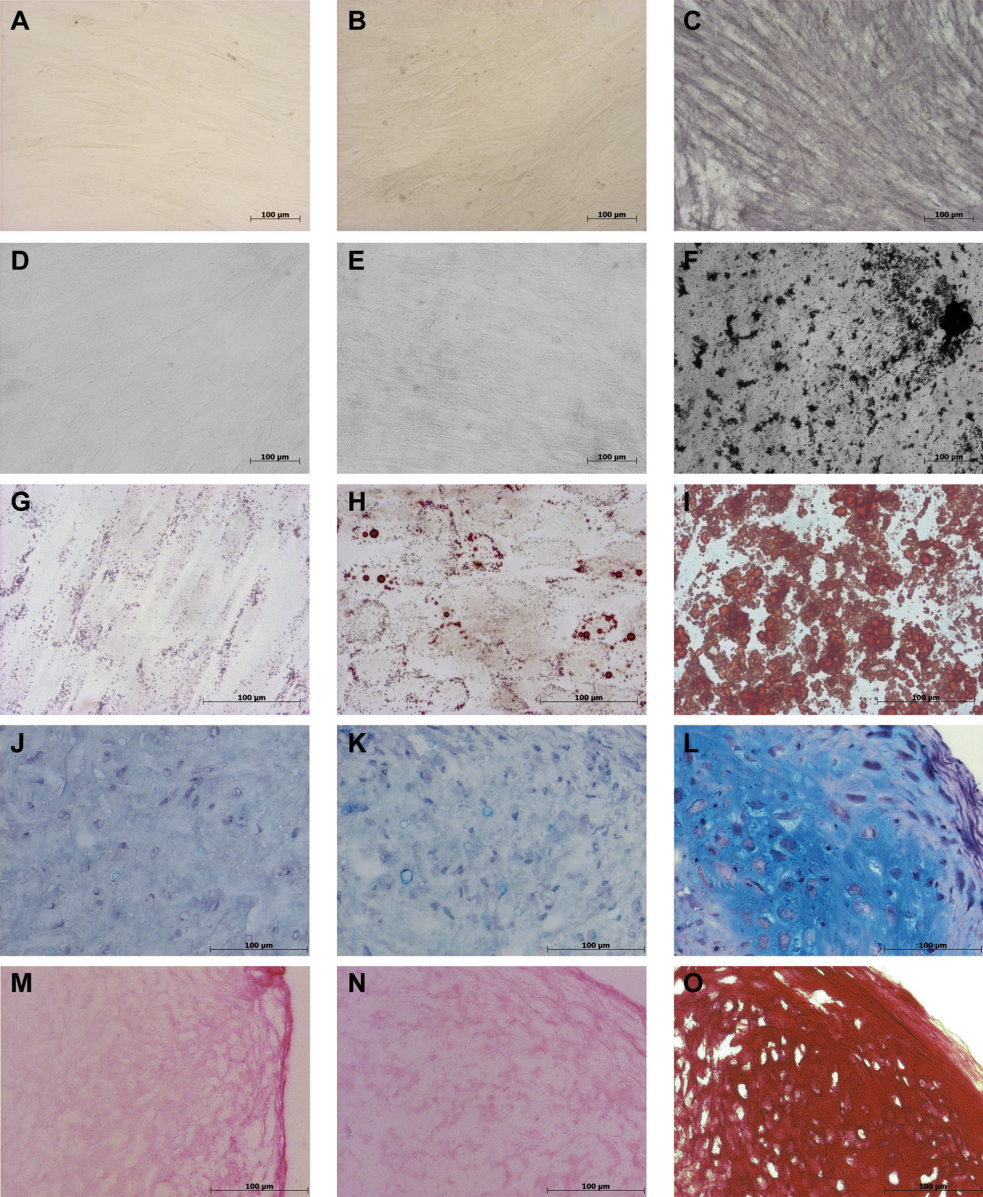
**Differentiation potential of human umbilical cord artery derived cells (HUCAC) compared with mesenchymal stem cells (MSC).** Multilineage differentiation potential of HUCAC from passage 2 (n = 3), passage 4 (n = 3) and MSC (positive control) from passage 2 (n = 1), passage 4 (n = 1) was qualitatively analyzed by histological and immunohistochemical staining methods. Osteogenic differentiation potential was studied by histological Von Kossa staining: **A**) unstimulated HUCAC (day 28), **B**) stimulated HUCAC (day 28), **C**) stimulated MSC (day 28) and histological alkaline phosphatase staining: **D**) unstimulated HUCAC (day 28), **E**) stimulated HUCAC (day 28), **F**) stimulated MSC (day 28). Adipogenic differentiation potential was studied by histological Oil Red O staining: **G**) unstimulated HUCAC (day 25), **H**) stimulated HUCAC (day 25), **I**) stimulated MSC (day 25). Chondrogenic differentiation potential was studied by histological Alcian PAS staining: **J**) unstimulated HUCAC (day 28), **K**) stimulated HUCAC (day 28), **L**) stimulated MSC (day 28) and by immunohistochemical collagen type II staining: **M**) unstimulated HUCAC (day 28), **N**) stimulated HUCAC (day 28), **O**) stimulated MSC (day 28).

To extend characteristic analysis for HUCAC, differentiation studies were performed to analyze whether the cells have the potential to differentiate into other cell types. Differentiation potential was examined in passage 2 and passage 4 fresh cultivated HUCAC and MSC from bone marrow (positive controls). Increased alkaline phosphatase activity and bone-specific calcified matrix areas (Von Kossa staining) were not detected for stimulated HUCAC compared to MSC (Figure 7 A, B, C and D, E, F). Furthermore, cartilage-specific collagen II and proteoglycans (Alcian PAS staining) were not detected for stimulated cells (Figure 7 J, K and M, N). By contrast, MSC have the potential to differentiate into chondrocytes, because red stained collagen II and blue stained proteoglycans were apparent (Figure 7 L, O). Controversial staining results with Oil Red O were obtained for adipogen-stimulated HUCAC, because 2 of 6 samples showed few enlarged lipid vacuoles (Figure 7 G, H, I), whereas no significant difference in number and size of adipose tissue-specific lipid vacuoles was detected for unstimulated and stimulated HUCAC in remaining samples. Altogether, multilineage differentiation potential of stimulated HUCAC was not demonstrated uniformly.

## Discussion

The human umbilical cord represents a potential cell source for cardiovascular tissue engineering because it is easily available after delivery without additional surgical intervention for the patient, ethically acceptable and offers adequate tissue mass. Cells derived from human umbilical cord vessels became of interest for cardiovascular tissue engineering since few years [12,17]. The aim of the recent study was to develop first essential strategies for establishing an individual cell bank consisting of vascular cells from the human umbilical cord under GMP conditions.

Adaption from R&D starting materials to GMP compliant starting materials was studied because only starting materials produced conforming to GMP are allowed to be used for the fabrication of cell products and medicinal products for subsequent clinical applications. Consequently, already established R&D protocols for cell isolation and cultivation were remodelled concerning to starting materials. Sufficient numbers of harvested HUCAC and HUVEC from primary cultures demonstrated that the cell isolation process was successful using GMP starting materials and resulted in isolation of similar cell numbers as using R&D protocols. Cell morphologies were monitored during primary cultures and expression of cellular marker molecules was analyzed after cell isolation in order to test whether desired cell types are obtained. Subsequently, fresh harvested cells from primary cultures (passage 0) were used for cryopreservation. After the cryopreservation process, cell viability, cell morphology, expression of cellular markers and proliferation potential were studied in order to analyze whether the cells maintain their characteristics and, finally, whether they are still suitable for intended application for a vascular cell bank.

Sufficient levels of viability were determined for harvested HUCAC (84.4 ± 9.4 %) and HUVEC (86.3 ± 8.6 %) from primary culture. Cell viability of cryopreserved cells was decreased directly after the thawing process, but already increased in passage 1 and achieved the viability of fresh cultivated cells. It was found that the short-term and long-term cryopreservation process did not influence cell viability or cell morphology with long-lasting impact. In morphological study, fresh cultivated and cryopreserved HUCAC demonstrated an elongated spindle-shaped morphology, also demonstrated in previous studies [16,17]. Endothelial cell-specific cobblestone like morphology observed for HUVEC in the present study was similarly demonstrated for HUVEC in a study by Ulrich-Merzenich et al. [29].

Cytoskeletal filament protein ASMA and cell surface protein CD90 were highly expressed by fresh cultivated HUCAC (control group). Correspondingly, high CD90 and ASMA expression was also described for fresh isolated human umbilical cord artery derived cells in studies by Schäfermeier et al. [15]. In the present study, expression of CD90 and ASMA was tested for recultivated short- and long- term cryopreserved cells, showing no significant difference in expression compared to fresh cultivated HUCAC. Both markers were also analyzed for cryopreserved myofibroblasts from human umbilical cord by immunofluorescence staining in previous studies [16,17]. Besides, here we demonstrated that nearly every single HUCAC expressed fibroblast-specific CD90 *and* smooth muscle cell-specific ASMA simultaneously. Smoothelin, an exclusively expressed marker in fully differentiated contractile smooth muscle cells [30], was not detected for HUCAC. Positive signals were also detected for extracellular matrix proteins such as collagen types I and III, elastin and for the glycoprotein fibronectin, proving that HUCAC are able to produce ECM components also found in native cord artery cross sections. These three last mentioned aspects suggest a high similarity of HUCAC, isolated with GMP compliant starting materials in the present study, to myofibroblasts - an intermediate cell type between smooth muscle cells and fibroblasts. These findings correspond to studies by Schäfermeier et al. and simultaneously showed that HUCAC are similar to pulmonary heart valve interstitial cells and therefore present a potential cell source for cardiovascular tissue engineering [15]. Furthermore, HUCAC are highly positive for CD44, CD166, CD105, CD73, CD29 and CD90 as well, also known as markers of MSC [31]. This is attributed to their common mesenchymal origin because stromal cells such as fibroblasts and myofibroblasts as well as bone marrow derived stromal stem cells are cell components of the *lamina propria*, a thin layer of loose connective tissue [32]. Covas et al. analyzed subcultivated mesenchymal stromal cells from diverse human tissue and confirmed similar marker profiles of e.g. skin fibroblasts (sFB) and MSC from bone marrow, concerning the expression of CD73, CD90, CD29, CD44 and CD166 amongst others [33]. Present differentiation experiments revealed that HUCAC do not have the potential to differentiate into the osteogenic, adipogenic and chondrogenic lineage, unlike MSC from bone marrow [31]. Finally, HUCAC shared the marker profile of MSC but they do not have multipotent differentiation properties indicating that HUCAC are more differentiated cells with a more restricted differentiation potential. Expression of cellular marker molecules was influenced by the cryopreservation process but not fundamentally. The subsequent recultivation process showed increased expression levels of cryopreserved HUCAC with no significant differences to fresh cultivated cells and accordingly no loss of their phenotypic characteristics.

Endothelial cell-specific markers such as CD31, CD146, CD105, vWF and eNOS were expressed by fresh cultivated HUVEC (control group), as confirmed by flow cytometry analysis and immunofluorescence staining. Existence of CD31 positive cells was observed in the endothelial lumen lining of native umbilical cord vein cross sections. As expected, fresh cultivated cells for example from passage 3 were highly positive for CD31 (99.1 ± 0.12 %) and also showed strong positive signals in immunofluorescence studies. Almost similar high proportions of CD31-positive HUVEC from the umbilical cord were also detected in flow cytometry studies by Covas et al. (86 %, passage 3) and in immunofluorescence studies by Schäfermeier et al. [15,33]. In the present study, expression level of CD146- positive HUVEC was considerable higher (passage 3: 99.8 ± 0.12 %) compared to the expression levels of flow cytometry in studies by Covas et al. (37 %, passage 3) [33]. Schugar et al. reported a high percentage of CD105 expressing cells (100 %) in the endothelial lumen of cord vessels, quantified by immunostaining of native human umbilical cord cross sections [34]. In the current study, similar large amounts of CD105-positive fresh cultivated HUVEC, also isolated from the endothelial lumen of cord vein, were found using flow cytometry analysis. High CD144-expression levels of about 100 % were measured in the endothelial lumen of vessels from cross sectioned native umbilical cords by Schugar et al. [34]. Fractions of CD144-positive fresh cultivated cells were also shown by flow cytometry analysis in the present study, although expression levels were a little lower compared to CD31, CD146 and CD105. HUVEC from the endothelium of native umbilical cord vein cross sections, detected by staining with anti-CD31 and anti-vWF, were recovered and analyzed in cell culture again, proving that cell isolation according to GMP compliant starting materials was precise and pure. The cryopreservation process influenced expression of cellular markers, but not with lasting impact for CD31, CD146 and CD105, because expression levels of fresh cultivated cells were achieved rapidly during the recultivation process, meaning that cryopreserved HUVEC did not lose their phenotypic characteristics. Only expression of CD144 was still affected in recultivated cryopreserved cells, as evident in flow cytometry studies. Contrarily, notable CD144-positive signals were detected for recultivated cryopreserved HUVEC from passage 3 and the signal intensity did not differ significantly from that of fresh cultivated cells.

Excellent proliferation properties were determined for fresh cultivated HUCAC, similarly demonstrated in proliferation studies of myofibroblasts like cells isolated from umbilical cord artery by Kadner et al. [8]. Proliferation potential of fresh cultivated and cryopreserved HUCAC was comparable without significant differences, as also reported in previous studies [16]. It was apparent that the long-term cryopreservation process influenced the proliferation potential of HUCAC (group B) concerning the extended lag phase. However, notable exponential growth was detected for long-term cryopreserved cells (group B), even though marginally delayed. An extended cultivation period will ensure cell numbers comparable to those of fresh cultivated HUCAC on day 7. Sufficient proliferation potential was also demonstrated for HUVEC. After an evident lag phase, exponential growth was observed from day 1 to day 4 for fresh cultivated (control group) and short-term cryopreserved cells (group A) leading to a stationary phase from day 4, as similarly described in previous proliferation studies of subcultivated HUVEC with related initial seeding densities [35]. In comparison, the lag phase of long- term cryopreserved cells (group B) was extended and the following exponential growth phase was delayed in starting, indicating that the cells required more time to adapt to the environmental conditions than short-term cryopreserved cells. Nevertheless, no significant difference in proliferation was determined on day 4 and 7 for group B-HUVEC compared to the control group, indicating that long-term cryopreserved cells would be suitable for cardiovascular tissue engineering.

The analysis of morphology and expression of cellular markers confirmed that the isolation process of HUCAC and HUVEC was successful using GMP compliant starting materials. Both cell types expressed their cell-specific markers and demonstrated typical expected morphologies. Sufficient viabilities of fresh isolated HUCAC and HUVEC suggest unhesitating application of these cells for future cell banking. Cryopreserved cells, involving directly thawed as well as recultivated cells, mostly maintain the phenotypic and functional characteristics of fresh non-cryopreserved cells. Furthermore, low standard deviation values of recultivated cryopreserved cells concerning proliferation and expression of cellular markers indicated great purity of the obtained HUCAC and HUVEC culture. Altogether, cells treated with GMP compliant starting materials did not change their phenotypic or functional characteristics, the same as cells treated with R&D starting materials. The establishment of an individual human vascular cell bank under GMP conditions would be feasible by using these cells.

On the basis of the recent study, we evaluated and established some methods to control the quality and identity of vascular umbilical cord cells which is a fundamental step for fully GMP compliant production. Thus, a specific cellular marker expression profile was established for HUCAC and HUVEC using flow cytometry analysis, applicable as quality control for determination of identity before cryopreservation and future storage in the cell bank. Standardized protocols taken as a basis for standard operating procedures (SOP) were generated for the entire *in vitro* fabrication process for detailed description of each single working step within cell isolation, cell cultivation, cell harvest and cyopreservation process, ensuring accuracy and reproducibility for intended validation procedures.

Certainly, the granting of a GMP manufacturing authorization according to §13 German Drug Act for banking of the described cell types needs further efforts, such as establishing additional quality controls for microbiological safety, purity and *in vitro* potency. The validation requires analytical methods according to European Pharmacopoeia / ICH Q2A/B, the qualification of umbilical cord procurement centers according to §20b German Drug Act including serological testing and a process validation according to annex 15 of the EU GMP Guideline.

Finally, for future therapeutic applications of cryopreserved HUCAC and HUVEC for tissue engineered cardiovascular constructs, the fabrication process of the constructs also has to be adapted to GMP conditions, because cell-based medicinal products (ATMP) for clinical trials have to be used in compliance with the principles of GMP, formulated in Commission Directive 2003/94/EC.

## Conclusion

The current study demonstrates first basic approaches which are important for the future establishment of a cell bank consisting of vascular cells from the human umbilical cord. Such a cell bank would offer great benefit especially for individual autologous therapeutic applications in terms of cell-based ATMP such as tissue engineered cardiovascular constructs. The fabrication of a cell bank and the production of cell-based ATMP for clinical applications have to take place in compliance with the principles of GMP to ensure safety and quality of the product.

Consequently, the cell isolation, cultivation and cryopreservation process were performed according to the GMP guidelines in the recent study. Sufficient numbers of cells with acceptable homogenous expression of cellular marker molecules were obtained, confirming that adaption from R&D to GMP compliant starting materials was successful. Continuative studies revealed that cryopreservation influenced cell viability, cellular marker expression and proliferation potential but without lasting impact - an important aspect for intended prospective application as a potential ATMP for cardiovascular tissue engineering. The results of the current study confirmed that the application of vascular cells from the human umbilical cord is in principle possible for establishing an individual human vascular cell bank under GMP conditions.

## Competing interests

The authors declare that they have no competing interests.

## Authors’ contributions

CL and RH conceived of the study and participated in its design. KK and GS participated in the design of the study and supported it with expert know-how in the field of good manufacturing practice. KK additionally supported the flow cytometry studies. WH organized and allocated the umbilical cords after declaration of agreement from the parents. BP performed the experiments and wrote the manuscript. AR participated in the translation of the study. All authors read and approved the final manuscript.
